# Heavy metals content in some non-alcoholic beverages (carbonated drinks, flavored yogurt drinks, and juice drinks) of the Egyptian markets

**DOI:** 10.1016/j.toxrep.2019.02.010

**Published:** 2019-02-25

**Authors:** Gomaa N. Abdel-Rahman, Mohamed B.M. Ahmed, Bassem A. Sabry, Safaa S.M. Ali

**Affiliations:** a Department of Food Toxicology and Contaminants, National Research Centre, 33 El-Bohouth St., P.O. Box: 12622, Dokki, Cairo, Egypt; b Spectroscopy Department, Physics Division, National Research Centre, 33 El-Bohouth St., P.O. Box: 12622, Dokki, Cairo, Egypt

**Keywords:** Carbonated drinks, Yogurts, Juices, Heavy metals

## Abstract

Heavy metals are elements present in trace quantities in the environment and, in small concentrations, they play important roles for the living organisms yet it can cause toxicity if exceeded the recommended levels. Toxic metals contamination is an important environmental problem that was mainly manifested in the growing industrial cities where the levels in toxic metals exceeded the recommended levels leading to the increase of several health problems (that vary from memory disorder to carcinogenic diseases). The main sources of food contamination by toxic metals are the increase in petroleum fuels exhausts and the addition of fertilizers and metal-based pesticides during farming processes. Nowadays, the non-alcoholic drinks represent one of the highest consumption groups worldwide especially in the Middle East and Islamic countries. The current work aims to investigate the safety of some non-alcoholic beverages (carbonated drinks, flavored yogurt drinks and juice drinks) from toxic metal contamination in the Egyptian market. The study revealed that non-alcoholic beverage samples (juices, flavored yogurts and carbonated drinks) in the Egyptian market are mostly free of lead (Pb), cadmium (Cd) and chromium (Cr) contamination. On the other hand, the results of current beverage samples indicate that iron (Fe), manganese (Mn) and nickel (Ni), except Mn in juices and carbonated drinks, were presented in concentrations above the recommended permissible limits of both the World Health Organization (WHO) and Egyptian Ministry Health (EMH). Meanwhile, Cu was found in concentrations below the recommended permissible limits.

## Highlights

• The studied samples (juices, flavored yogurts and carbonated drinks) were mostly free of Pb, Cd and Cr.
• Concentrations of Fe, Mn and Ni were above the recommended permissible limits in most of the beverage samples.
• Canned beverages were most contaminated by toxic elements as compared to the plastic bottled beverages.

## Graphical abstract

**Figure:**
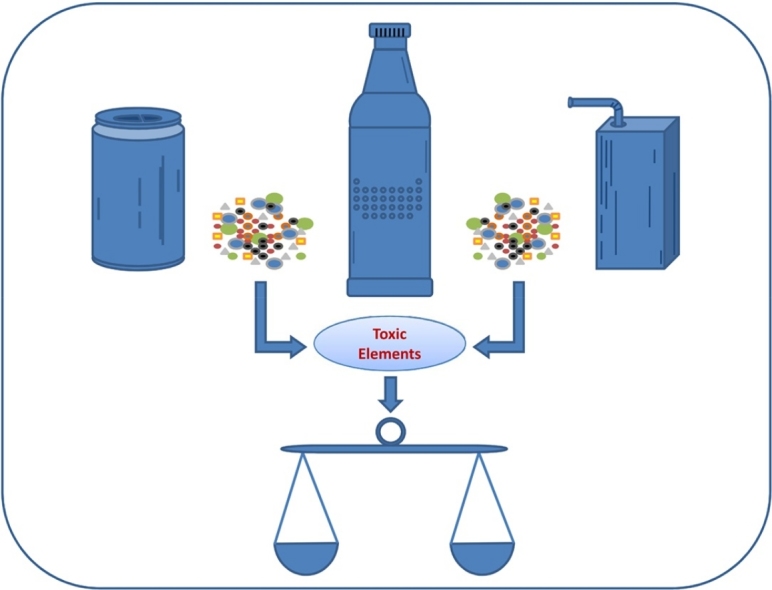

## 1 Introduction

The non-alcoholic beverages include many drinks such as carbonated drinks, juices, energy drinks, bottled water, coffee, tea and probiotic drinks. Carbonated drinks represent the highest portion of non-alcoholic consumed beverages. However, bottled water and juices are in the second and third grades, respectively [1]. Manufacturers of these beverages should require unusual caution to ensure the purity of constituents used in this industry such as raw sources of water and packaging material which mostly are the sources of contaminants in the beverages [2].

Carbonated drinks, flavored yogurt drinks and juices drinks are the most non-alcoholic beverages consumed by the Egyptian populations. The fruit juices are obtained by mechanical extraction (squeezing) of different fruits. The fruit juices contain many nutrients such as minerals, vitamins (especially vitamin C), antioxidants, carotenoids, phytochemicals and dietary fiber, which are essential for human health [3,4]. Constituents of the carbonated drinks are water, carbon dioxide and flavor [5]. Meanwhile, flavored yogurt drink is made from fermented milk, sugar, colorings and artificial or natural flavorings [6]. Yogurt drink is a superior source of protein, vitamins (D, B-6, B-12, niacin and riboflavin), potassium, calcium and phosphorus [7].

However, these beverages may contain toxic metals, which have a significant health impact for humans [8]. Some heavy metals such as Cu, Fe and Mn are important for human life, where these metals are coenzymes and natural essential substances for growth and respiration. In contrast, other metals such as Pb and Cd considered as very toxic contaminants and have no biological importance [9,10] and cause serious adverse health effects in the human body. In this respect, Flora et al. [11] and Matovic et al. [12] reported that Pb can be accumulated in erythrocytes and then replace Zn in δ-aminolevulinic acid dehydratase (an important enzyme in heme biosynthesis) and thus inhibits its function. Also, Buha et al. [13] and Buha et al. [14] noticed that Cd can induce carcinogenic diseases such as pancreatic cancer and thyroid cancer.

Heavy metals present in nature with trace concentrations can be taken into the human body by ingestion, inhalation and dermal absorption [15], then cause toxicity if exceeded the recommended doses. The accumulation and toxicity of metals in the human body depend on the chemical form. For example, about 15% of ingested inorganic Pb is absorbed, while about 80% of ingested organic Pb is absorbed [16]. Also, inorganic Hg is renal toxicants, in contrast, organic Hg is toxic to the nervous system [17].

Heavy metals and phthalates are considered the major detected contaminants in packaged food; specifically, beverages as confirmed by recent studies [18,19]. The main sources of beverages' contamination by toxic metals includes; the high content of toxic metals in the used fruits which may return to the contamination of agricultural soil and irrigation water [[20], [21], [22]], and the extreme use of pesticides and fertilizers during cultivation of fruits as well as fodder plants consumed by dairy animals [23,24]. As well, the contamination of water which used in the beverages production [25], besides the natural composition of raw materials which used in beverages production (water, milk, fruits and added sugar), packaging materials and used processing technologies [26].

So, in order to maintain human health, the levels of toxic metals need to be regularly monitored in many food materials in order to ensure its safety for human consumption. So that, the current work aims to determine levels of toxic metals contamination in some non-alcoholic beverage (carbonated drinks, flavored yogurt drinks and juice drinks) in the Egyptian market to provide some baseline information in this field in comparison to the permissible limits.

## 2 Materials and methods

### 2.1 Sampling

Random samples of flavored yogurt drinks, juice drinks and carbonated drinks were collected from Egyptian local markets as 12, 18 and 24 samples, respectively (samples were collected in three replicates). The sampling date was between January and June 2018.

### 2.2 Sample preparation

Samples of juice and yogurt drinks were digested by the dry-ashing process; 5 g of sample was weighed in crucibles and dried at 105 °C in an oven. The crucible was placed in the Muffle furnace (Vulcan 3-550, made in USA), the temperature degree was increased step wisely up to 550 °C and then left 8 h until the sample was completely digested. The crucible with ash was put in a desiccator to cool. The obtained ash was dissolved using 1 ml HCl conc. and transferred by de-ionized water to complete a volume of 25 ml [27]. The ash suspension was filtered through ashless filter paper Whatman No. 42 and stored in a refrigerator until the determination by Inductive Coupled Plasma Optical Emission Spectrometry (ICP-OES).

Wet digestion method was used for digestion of carbonated drinks samples according to Godwill et al. [28]. Briefly, a 30 ml carbonated drink sample was placed in a rotary flask to evaporation of its gases. Subsequently, 10 ml of nitric acid (69%) was added to 10 ml of the sample and the mixture was evaporated on a hot plate in a fume cupboard until the brown fumes disappear leaving white fumes. Distilled water was added to make up the volume to 25 ml which was then filtered and ready for analysis by ICP-OES.

### 2.3 Standards

Standard solutions of studied heavy metals *i.e.* lead (Pb), cadmium (Cd), chromium (Cr) copper (Cu), iron (Fe), manganese (Mn) and nickel (Ni) were provided by Merck (Darmstadt, Germany). The standards were prepared from the individual 1000 mg kg^−1^ standards (Merck), in 0.1 N HNO_3_. Working standards were prepared from the previous stock solutions by dilution using 0.1 N HNO_3_ till the needed concentrations for determination [29].

### 2.4 Heavy metals analysis

Analysis for investigated heavy metals was performed at Water Pollution Department, National Research Centre using the Agilent 5100 Synchronous Vertical Dual View (SVDV) ICP-OES according to APHA [30], with Agilent Vapor Generation Accessory VGA 77. For each series of measurements, the intensity calibration curve was constructed composed of a blank and three or more standards from Merck Company (Germany). Accuracy and precision of the Fe, Mn, Cd, Pb, Ni, Cr and Cu ions measurements were confirmed using external reference standards from Merck, and standard reference material and quality control sample from National Institute of Standards and Technology (NIST), were used to confirm the instrument reading.

### 2.5 Statistical analysis

Results were subjected to one-way analysis of variance (ANOVA) of the general linear model (GLM) using SAS [31] statistical package. The results were the average of three experiments (p ≤ 0.05).

## 3 Results and discussion

### 3.1 Heavy metal analysis

#### 3.1.1 Carbonated drinks

Concentrations of different elements in carbonated drinks is demonstrated in Table 1 which revealed that, 100% of the tested samples were free of Pb, Cd and Cr, and that was on the contrary to previous reports that showed high incidence ratios in the samples which varied between 5–20% [2] and 40–70% [32]. Also, Ni and Mn were below detection limits in most of the samples except for Ni in Mirinda and Mn in Pepsi. These results are comparable to those obtained by Woyessa et al. [33] who indicated that Ni not detected in all soft drink samples, while Mn was only detected in two samples of soft drink (Fanta and Sprite). The variation in the levels of contamination could obviously be attributed to the difference of the source of raw materials and manufacturing process condition that vary from a factory to another and from a country to another.

**Table 1:** Concentration of different trace elements in carbonated drinks.

Metals (mg kg^−1^)	Pepsi		Fanta		Sprite		Mirinda		LSD
	Plastic	Cans	Plastic	Cans	Plastic	Cans	Plastic	Cans	
**Copper**	0.16 ^b^ ± 0.01	0.21 ^a^ ± 0.02	0.06 ^c^ ± 0.01	0.10 ^c^ ± 0.02	0.07 ^c^ ± 0.01	0.09 ^c^ ± 0.01	<d.l.	0.09 ^c^ ± 0.02	0.04
**Iron**	19.71 ^b^ ± 0.89	31.63 ^a^ ± 1.01	1.56 ^d^ ± 0.12	2.72 ^d^ ± 0.16	1.29 ^d^ ± 0.04	1.52 ^d^ ± 0.05	14.01 ^c^ ± 0.86	20.34 ^b^ ± 0.9	2.12
**Nickel**	<d.l.	<d.l.	<d.l.	<d.l.	<d.l.	<d.l.	0.18 ^b^ ± 0.01	0.24 ^a^ ± 0.02	0.03
**Lead**	<d.l.	<d.l.	<d.l.	<d.l.	<d.l.	<d.l.	<d.l.	<d.l.	–
**Cadmium**	<d.l.	<d.l.	<d.l.	<d.l.	<d.l.	<d.l.	<d.l.	<d.l.	–
**Chromium**	<d.l.	<d.l.	<d.l.	<d.l.	<d.l.	<d.l.	<d.l.	<d.l.	–
**Manganese**	0.04 ^b^ ± 0.01	0.12 ^a^ ± 0.02	<d.l.	<d.l.	<d.l.	<d.l.	<d.l.	<d.l.	0.03

<d.l.: below the detection limit.

Means followed by different subscripts within row are significantly different at the 5% level.

In contrast, Cu and Fe were detected in all carbonated drinks with different concentrations, where Cu concentrations ranged between 0.06 and 0.21 mg kg^−1^ and its highest concentration was found in Pepsi drink. Nevertheless, the carbonated drink samples were superabundant with Fe element which ranged from 1.29 mg kg^−1^ in Sprite (plastic bottles) to 31.63 mg kg^−1^ in Pepsi (cans bottles). These results are close to those obtained by Bingol et al. [34] who noticed that concentrations of Cu element in carbonated drinks ranged from 0.03 to 0.13 mg kg^−1^. Also, Ofori et al. [35] revealed that Fe element had the highest level in carbonated drink sample as compared to other determined metals.

Generally, concentrations of Cu, Fe, Ni and Mn were higher in cans bottles than in plastic bottles. Also, the results obtained by Francisco et al. [36] indicated that the metals concentration was higher in the canned carbonated drinks than in the plastic bottled carbonated drinks. This finding supports the assumption that the metals may migrate from the packaging materials to the packaged food or drink [37].

The variation in metals content between our tested samples and previous studies may be returned to the type of water used during the production of carbonated drinks in Egypt (groundwater or River Nile water). In this respect, Bassioni et al. [38] reported the presence of Cu, Fe, Ni and Mn in Egyptian groundwater being 0.21, 1.06, 0.04 and 0.03 mg kg^−1^, respectively. Meanwhile levels of the same metals in River Nile water were in the ranged of (0.011–0.024 mg kg^−1^) for Cu, (0.047–0.463 mg kg^−1^) for Fe, (0.008–0.025 mg kg^−1^) for Ni and (0.038–0.360 mg kg^−1^) for Mn as reported by El-Bouraie et al. [39]. Also, the level of toxic metals in carbonated drinks depends on the efficiency of purification processes during production steps [28].

#### 3.1.2 Juices

Data in Table 2 indicated that Pb, Cd and Cr were non-detectable in all juice samples, while Cu content varied between 0.17 and 0.56 mg kg^−1^ in the tested samples. Oriental hibiscus recorded the highest significant levels of Fe (43.88 mg kg^−1^), Ni (0.53 mg kg^−1^) and Mn (1.24 mg kg^−1^). In contrast, Oriental carob, guava juice and peach juice had the lowest values of Fe (3.28 mg kg^−1^), Ni (0.15 mg kg^−1^) and Mn (0.12 mg kg^−1^), respectively. The metal variability between the different juice samples may be resulted from the raw materials and water used in the juices production, the conditions of plant growing such as levels of toxic metals in soil and irrigation water, the environmental contamination (fertilizers and pesticides), purity of the added sugar, and the industrial processing and contamination from containers [26].

**Table 2:** Concentration of different trace elements in fruit juices and oriental drinks.

Metals (mg kg^−1^)	Cappy mango	Cappy peach	Fresh mango	High fresh guava	Oriental hibiscus	Oriental carob	LSD
**Copper**	0.24 ^cd^ ± 0.02	0.17 ^de^ ± 0.01	0.28 ^c^ ± 0.02	0.38 ^b^ ± 0.02	0.15 ^e^ ± 0.03	0.56 ^a^ ± 0.05	0.09
**Iron**	31.59 ^b^ ± 0.79	11.66 ^d^ ± 0.41	3.48 ^e^ ± 0.11	23.04 ^c^ ± 0.23	43.88 ^a^ ± 0.84	3.28 ^e^ ± 0.22	1.78
**Nickel**	0.23 ^c^ ± 0.02	0.16 ^d^ ± 0.01	0.39 ^b^ ± 0.02	0.15 ^d^ ± 0.01	0.53 ^a^ ± 0.01	0.36 ^b^ ± 0.01	0.06
**Lead**	<d.l.	<d.l.	<d.l.	<d.l.	<d.l.	<d.l.	–
**Cadmium**	<d.l.	<d.l.	<d.l.	<d.l.	<d.l.	<d.l.	–
**Chromium**	<d.l.	<d.l.	<d.l.	<d.l.	<d.l.	<d.l.	–
**Manganese**	0.24 ^c^ ± 0.03	0.12 ^d^ ± 0.02	0.32 ^c^ ± 0.03	0.41 ^b^ ± 0.01	1.24 ^a^ ± 0.04	0.14 ^d^ ± 0.02	0.08

<d.l.: below the detection limit.

Means followed by different subscripts within row are significantly different at the 5% level.

These results are comparable to those reported by Hassan et al. [40] who noticed that, Pb and Cd were not detected in the most samples of fruit juices. They stated that, the mango juice had the highest concentration of Fe as compare to peach and guava juices and this finding was similar to the present study. Also, Abdel-Rahman and Abdellseid [9] studied the levels of Pb, Cd, Fe and Cu in Libyan mango juice which were very close to those of the present results except for Fe levels. While, Farid and Enani [41] reported lower levels of Fe, Mn and Ni in Saudi Arabian mango juice than those in the present study. Other reports revealed higher levels of Pb and Cd in Saudi Arabian juices [42].

Moreover, another report by Khan et al. [43] summarized those toxic metals concentrations in some tested juices. Cd level was below the detection limit in mango and peach juices, while Cu and Ni were detected in 24% of the samples. While, Ajai et al. [44] noticed that, Pb, Cd, Cr and Mn were not detected in guava and mango juices, whilst Fe and Cu were detected and its concentrations were similar to the current study. In another report where the toxic metals were determined in peach juice by Dehelean and Magdas [45], the results revealed that Pb, Cd and Cr were detected in all analyzed samples, which conflicted with the present study, while the levels of Cu and Mn were similar to this study. Mango, guava and peach juices were included in previous studies, because it is the most favorable and consumable in the Middle East. But, oriental juices (hibiscus and carob) were not included in the previous studies. So, it was not possible to find similar reports discussing toxic metal concentrations in such drinks and no available data for comparison with the current results.

#### 3.1.3 Flavored yogurt drinks

The obtained results in Table 3 revealed that Pb, Cd and Cr in the samples of flavored yogurt drinks were below the detection limits. However, Cu content varied between 0.17 and 0.23 mg kg^−1^ in the tested samples. Significant variations were observed for Fe content in different drinks of flavored yogurt as it reached as high as 8.59 mg kg^−1^ in mango yogurt drink and as low as 3.17 mg kg^−1^ in strawberry yogurt drink. The peach yogurt drink recorded the highest significant Ni levels reaching 1.37 mg kg^−1^ compared to mulberry yogurt drink that the Ni content was found to be 0.23 mg kg^−1^, the same trend was also observed in the Mn in peach and mulberry yogurt drinks recording 0.12 and 0.06 mg kg^−1^, respectively.

**Table 3:** Concentration of different trace elements in flavored yogurt drinks.

Metals (mg kg^−1^)	Yogurt with Mango	Yogurt with Peach	Yogurt with Strawberry	Yogurt with Mulberry	LSD
**Copper (Cu)**	0.23 ^a^ ± 0.02	0.22 ^a^ ± 0.025	0.17 ^b^ ± 0.01	0.17 ^b^ ± 0.01	0.04
**Iron (Fe)**	8.59 ^a^ ± 0.22	5.27 ^b^ ± 0.02	3.17 ^d^ ± 0.06	4.60 ^c^ ± 0.15	0.54
**Nickel (Ni)**	0.51 ^b^ ± 0.04	1.37 ^a^ ± 0.12	0.40 ^bc^ ± 0.04	0.23 ^c^ ± 0.02	0.25
**Lead (Pb)**	<d.l.	<d.l.	<d.l.	<d.l.	–
**Cadmium (Cd)**	<d.l.	<d.l.	<d.l.	<d.l.	–
**Chromium (Cr)**	<d.l.	<d.l.	<d.l.	<d.l.	–
**Manganese (Mn)**	0.10 ^ab^ ± 0.01	0.12 ^a^ ± 0.01	0.09 ^b^ ± 0.01	0.06 ^c^ ± 0.01	0.02

<d.l.: below the detection limit.

Means followed by different subscripts within row are significantly different at the 5% level.

Tarakcl and Dag [46] reported that the levels of Pb, Cd, Cr, Cu and Mn in yoghurt samples were higher than those in the present findings, but Fe and Ni concentrations were lower than our current values. Malhat et al. [47] studied heavy metals in Egyptian cow's milk and they found higher Fe, Cu, Pb and Cd levels than those of the present study. Also, Issa et al. [48] reported that the levels of Pb, Cd, and Cu in Egyptian yoghurt were higher than ours. Meshref et al. [49] studied toxic metals in some Egyptian dairy products. They found that the levels of Fe and Cu were comparable to ours of the present study, whereas Pb and Cd were higher. Moreover, Pb and Cd were detected in flavored milk collected from Qena city, Egypt [6].

The variation of toxic metal concentrations in samples of flavored yogurt drinks may be returned to some factors such as type of fruits used as natural flavorings [50], locality of milk, levels of toxic metal contamination in the feed of dairy animals, season of production and may also be a result of handling contamination through milk transportation [51]. In this respect, El-Sayed et al. [52] assessed the levels of Fe, Zn, Cu, Cr, Pb and Cd in raw cow milk samples collected from different Egyptian regions during different periods of the year 2009. They reported that Shubra samples had the highest levels of Cd, Cr, Fe and Cu, while Menofia and Tanash samples had the highest levels of Pb and Zn, consecutively. They observed that the highest levels of Cd, Cr and Zn were recorded during January–February period, while the highest levels of Pb, Cu and Fe were recorded during May–June period. Also, El-Gendy et al. [53] noticed that levels of iron in milk samples which collected during warm or hot months were higher than those collected in cold months.

### 3.2 Heavy metals in the present investigation as compared to the recommended drinking water standards

Lately, the prevalence of human diseases such as chronic anemia, liver cirrhosis and renal failure were increased as a result of increasing toxic metal contamination. The consumption of non-alcoholic beverage was markedly increased, so the safety of such drinks became a major concern as it may represent sources of human exposure to toxic elements.

So, the toxic metal levels in the present investigated samples were compared with the recommended maximum permissible limits (MPL) of metals in drinking water as given by the World Health Organization (WHO) and Egyptian Ministry Health (EMH). The results in Table 4 showed that the average concentrations of Cu, Cr, Pb and Cd in all nonalcoholic beverage samples, as well as Mn in juices and carbonated drinks were within the maximum permissible limits of toxic metals in drinking water [54,55]. But, the average concentrations of Fe and Ni in all tested samples, as well as Mn in samples of flavored yogurt drinks exceeded the maximum permissible limits specified by WHO and EMH.

**Table 4:** The average concentrations of heavy metals in Egyptian non alcoholic beverages as compare with maximum permissible limit of different international organizations in drinking water.

Items	Heavy metals (mg kg^−1^)						
	Cu	Fe	Mn	Ni	Cr	Pb	Cd
**Yogurt fruit juices**	0.194	5.41	0.87	0.625	<d.l.	<d.l.	<d.l.
**Fruit juices**	0.294	19.485	0.41	0.300	<d.l.	<d.l.	<d.l.
**Carbonated drinks**	0.094	11.6	0.02	0.525	<d.l.	<d.l.	<d.l.
**Permissible limit of WHO (2011)**	2.0	0.3	0.4	0.07	0.05	0.01	0.003
**Permissible limit of EMH (2007)**	2.0	0.3	0.4	0.02	0.05	0.01	0.003

Although, the levels of Fe in the analyzed samples were above the safe limit of Fe in drinking water (0.3 mg kg^−1^) according to WHO [54], these levels were below the iron requirement for the human body (10–50 mg per day) as recommended by FAO [56]. These high concentrations of Fe in the analyzed samples may be returned to the release of Fe element from the metallic containers which used in the preparation of flavored yogurt drinks, juice drinks and carbonated drinks [35,57] or because of the normal existence of Fe in the raw material used in the production [36].

The results in Table 4 revealed that nonalcoholic beverage samples (juices, yogurts and carbonated drinks) in the Egyptian market are mostly free of Pb, Cd and Cr contamination. The highest averages of Cu and Fe levels were observed in juice samples, while the highest averages of Mn and Ni levels were found in samples of the flavored yogurt drinks. These results are in parallel with those recorded by Ofori et al. [35] who stated that levels of Cu and Fe in fruit juice were higher than its levels in carbonated drinks. Also, Ameyaw et al. [58] reported that the trace elements in the fruit juices were found to be more than those of the carbonated beverages.

## 4 Conclusion

Toxic metals represent a major threat to human health. Although Pb, Cd and Cr were absent in the studied samples, Ni, Fe, Mn and Cu were presented in all the tested samples of nonalcoholic beverages. Most of the detected metals were above the permissible limits in water. Therefore, it is very important to follow a food safety system during the manufacturing of nonalcoholic beverages in order to avoid high levels of toxic metals, and also to set a permissible limit for each metal in juices, flavored yogurt drinks and carbonated drinks. Finally, it is recommended to regularly check the raw materials of the nonalcoholic beverages as well as the processing procedures to reduce the transfer of these toxic metals in final products as much as possible.

## Transparency document

Transparency Document
